# Development of an Adapted Model for Decision-Making to Improve Reasoning and Risk Assessment in an Emergency Team: A Prospective Simulation Study

**DOI:** 10.3390/medicina55070339

**Published:** 2019-07-04

**Authors:** David Häske, Wolfgang Dorau, Niklas Heinemann, Jan-Philipp Stock, Benjamin Schempf

**Affiliations:** 1 German Red Cross, Emergency Medical Service, 72764 Reutlingen, Germany; 2 University Hospital, Eberhard Karls University Tübingen, 72076 Tübingen, Germany; 3 Department of Anesthesiology, Intensive Care Medicine, Emergency and Pain Medicine, Klinikum am Steinenberg, 72764 Reutlingen, Germany; 4 Department of Internal Medicine, Cardiology, Angiology and Intensive Care Medicine, Klinikum am Steinenberg, 72764 Reutlingen, Germany

**Keywords:** decision-making, risk assessment, simulation training, paramedic, medical education

## Abstract

*Background and Objectives:* Medical staff is rarely trained in structured decision-making, relying instead on intuition without due consideration for the associated pros and cons. *Materials and Methods:* We adopted a model for decision-making to improve reasoning and risk assessment and carried out a prospective simulation study using paramedic students in a three-year training program. We conducted a training session in which participants were lectured on decision-making using the FAR-BEK model (German abbreviation for facts, alternatives, risks, competence, decision, control), physiological processes in decision-making under stress, as well as medico-legal aspects for the comprehension and justification of medical decisions. We analyzed pre- and post-training scenarios to elucidate the influence of training on decision-making. *Results:* Twenty paramedic students, with a mean age of 22.0 ± 1.7 years, took part in the study. The question of whether decision aids can be applied, initially affirmed by 40% of participants, rose to 71.4% (*p* = 0.011) following our training. Confidence in decision-making increased on a 7-point Likert scale from 4.5 to 4.8 points (*p* < 0.394). The reasoning behind the decisions rose from 5.3 to 5.6 points (*p* < 0.081). Indication, options, and risks rose significantly, from 5.4 to 6.1 points (*p* = 0.045). Overall, our simulation training significantly increased the points of decision support taken into account (57.8% vs. 88.9%, *p* < 0.001). Viewed individually, the largest increase of 180% was seen in risk assessment (33.3% vs. 93.3%, *p* < 0.002). The second largest increase of 150% was seen in the question of one’s own permissions (26.7% vs. 66.7%, *p* < 0.066). Also, the control increased (40.0% vs. 86.7%, *p* < 0.021). *Conclusions:* With a brief training course, both the awareness and the implementation of a structured decision-making model in paramedic students can be significantly increased. Nevertheless, no definitive conclusions can be made with respect to the implementation of real patient care. The application of structured, standardized decision-making tools may need to be further consolidated in routine medical use.

## 1. Introduction

The impact of decisions made in emergency medicine remains higher than in most other medical fields [1]. In addition, the urgency places an increased pressure on emergent care staff, who must often make high-stakes decisions within seconds with incomplete information on the patient and their medical situation [2]. However, clear and purposeful thinking is only possible to a very limited extent when under significant stress and, as stress increases, the brain’s short-term memory capacity decreases to a few seconds [3]. The resulting decisions are subcategorized into two cognitive types in the dual process theory: intuitive-automatic and rational-analytic. The more urgently a decision must be made, the more the non-reflective, intuitive process becomes active [2].

If a decision-making process is more intuitive, two mechanisms play a major role: similarity matching, which typically arises from intuition, and frequency gambling, which focuses on the decision with which one previously had the most success. On the one hand, these so-called heuristics are quite effective and nearly automatic; on the other hand, they are prone to systematic errors as they neglect current information and unfamiliar events, ultimately leading to poor decisions [4].

To address this problem, structured decision-making methods have been used in aeronautics for many years and have long since gained recognition in medical fields, especially emergency medicine. Crisis Resource Management (CRM) includes behavioral principles designed to increase safety through prevention and management of critical situations while leveraging experiences in the aviation field. Furthermore, CRM decision-making content addresses the importance of communication, task management, situational awareness, and teamwork [5].

### Description of the Problem

Numerous clinical pathways can be undertaken in emergency medicine. Surprisingly, however, most German standard operation procedures (SOPs) do not involve a structured decision-making process despite the expectation of complex medical and legal decision-making, sometimes, in time-critical situations. Apart from CRM courses, medical staff is rarely trained in structured decision-making. In our training and simulations, we saw that decisions were only partially—or not at all—justified. Here, a “gut feeling” (i.e., intuition) was often used without due consideration of the associated pros and cons.

## 2. Materials and Methods

### 2.1. Study Design and Setting

We carried out a prospective simulation study, which took place at the training site of the German Red Cross Emergency Medical Service in Reutlingen, Germany, as part of a curricular training course. In addition to paramedics, emergency physicians are also deployed in emergency medical services in Germany, who are dispatched in the event of life-threatening illnesses, injuries, or other indications that exceed the competence of paramedics. Dispatch is coordinated by the dispatch center following an emergency call or upon request by paramedics.

### 2.2. Participants

All participants were paramedic students enrolled in a three-year training program. Students participated voluntarily and were asked to provide written informed consent. In their training, and within the study, the final competencies had already been practiced. The approved competencies of paramedics are remarkably heterogeneous in Germany despite state-specific training. For training, federal-state SOPs are applied and, for concrete implementation, further internal guidelines and quality assurance measures are applied. The local competencies at the place of study are shown in Table 1. For all measures surpassing these competencies, an emergency physician must be requested to the site.

### 2.3. Intervention

We held a training session in which participants were lectured on decision-making, physiological processes in decision-making under stress, and medico-legal considerations in order to enhance comprehension and proffer a justification for certain medical decisions. Theoretical examples were provided as supplementation. The content examples are in Table 2.

Central to the training was the FAR-BEK model as a structured decision-making tool. FAR-BEK was developed as part of a joint venture to create a framework for a competence system in the context of paramedic and patient safety. FAR-BEK is the German abbreviation for facts, alternatives, risks, competence/ability, decision, control (Table 3). FAR-BEK is based on—and shares similar features with—the Facts, Options, Risks and Benefits, Decision, Execution, Check (FOR-DEC) model used in aviation [6]. FOR-DEC, on the other hand, seemed to be very well suited as a template, as it is probably the best-known decision-making aid in the field of CRM and emergency medicine. Due to feedback from the staff, a German-language model appeared to be more effective, and the opportunity to introduce “ability” became apparent. The peculiarities, however, are the substantive agreement with the (German) criminal law § 34 of the “justified emergency” (necessity, proportionality, appropriateness) as well as “Ability/Authorization/Competence”; this raises the question, regardless of qualification, of whether the paramedic, or even the emergency physician, possesses the necessary competence for a procedure in a given situation. This relates to personal suitability (training status, performance, etc.) and formal approval, for instance.

### 2.4. Study of the Intervention(s)

We chose a pre- and post-comparison approach to see what influence the lecture, and the decision model presented therein, had on training scenarios. We determined the fulfillment of the 6 possible FAR-BEK points and compared them in the pre- and post-training analysis, and presented as the points of decision support taken into account by the participants. In addition, we conducted a pre- and post-training survey with a Likert scale (1–7 points ranging from “totally agree” to “totally disagree”) regarding the subjective assessment of decision-making.

### 2.5. Statistical Methods

Statistical evaluation was performed with the chi-square test for categorical variables. For the dependent samples, the *t*-test was used. A two-tailed test was considered statistically significant at *p* < 0.05. For continuous variables, data are shown as mean ± standard deviation. For categorical variables, percentages are presented. All data were analyzed using the statistical software SPSS (Version 25.0, IBM Inc., Armonk, NY, USA).

### 2.6. Ethical Considerations

All subjects provided informed consent for inclusion prior to participation. The study was conducted in accordance with the Declaration of Helsinki, and the protocol was approved by the Ethics Committee of the State Chamber of Physicians of Baden-Württemberg (Project ID RT-DRK-201903-1, approved on 15/03/2019).

## 3. Results

Twenty paramedic students took part in the study, with a mean age of 22.0 ± 1.7 years, and 35% (*n* = 7) of whom were female. In terms of prior knowledge, 50% of participants claimed knowledge of decision aids, and 50% also claimed familiarity with the FOR-DEC model. CRM or principles of CRM were indicated as familiar to 90% of participants. The question of whether decision aids can be applied, affirmed in 40% of participants, rose to 71.4% (*p* = 0.011) following the training.

**Figure 1:**
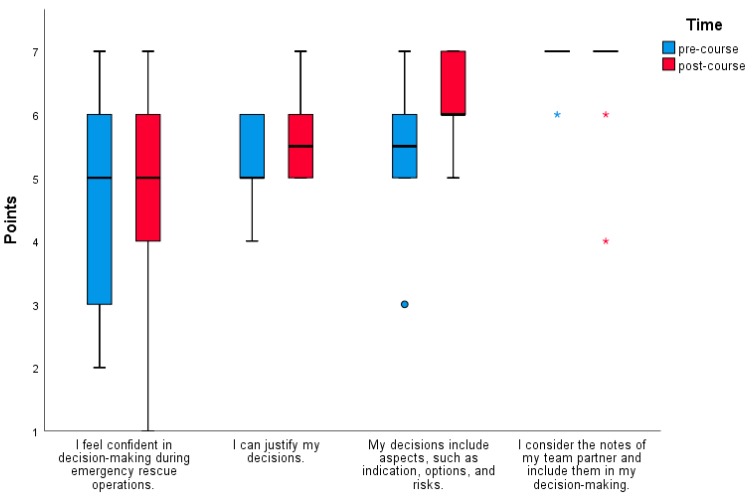
Results of the survey on the subjective assessment presented as boxplots with median, 25%, and 75% quartile. Whiskers represent interquartile range * 1.5.

There were no major changes in the subjective assessment before and after the training. The confidence in decision-making rose from 4.5 to 4.8 points (*p* < 0.394), and the reasoning of decisions from 5.3 to 5.6 points (*p* < 0.081). Aspects, such as indication, options, and risks, rose significantly, from 5.4 to 6.1 points (*p* = 0.045). To consider notes from my team partner and include them in the decision-making process remained unchanged and high (6.8 to 6.8 points, *p* = 0.555) (Figure 1).

**Figure 2:**
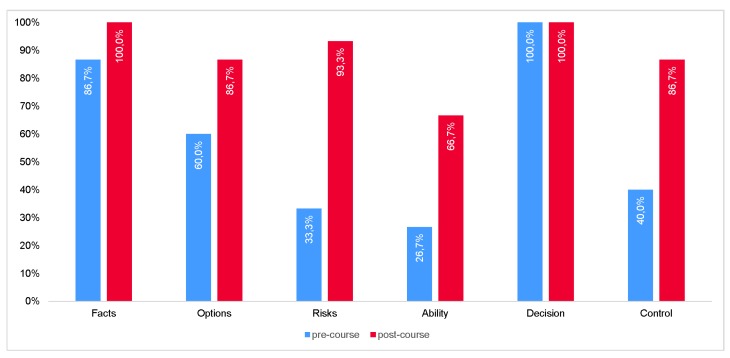
Pre- and post-course comparison of the use of the FAR-BEK (German abbreviation for facts, alternatives, risks, competence, decision, control) model (*n* = 20).

Overall, the points for decision support taken into account by participants had increased significantly in the simulation training following the lesson (57.8% vs. 88.9%, *p* < 0.001) (Figure 2). Viewed individually, the largest increase (180%) was seen in risk assessment (33.3% vs. 93.3%, *p* < 0.002). The second largest increase (150%) was seen in the question of one’s own abilities (26.7% vs. 66.7%, *p* < 0.066). Control also increased (40.0% vs. 86.7%, *p* < 0.021). It is of note that, although the facts have increased in quantitative terms (86.7% vs. 100%, *p* = 0.483), the quality of the facts also improved during observation of the training. The question of options also increased (60.0% vs. 86.7%, *p* = 0.215).

## 4. Discussion

In both medical rescue operations and various medical training programs, many decisions were seen to be made “from the gut”—in other words, by intuition. The value of such decision-making should not be dismissed per se; however, ‘gut feelings’ are often based on personal experience, and such decisions are often difficult to substantiate retrospectively. Furthermore, this approach is subject to inadequate consideration for potential consequences, such as medical and legal risks, given the retracted time-period permitted to the decision-maker in emergency situations. It is well-known that goal-oriented thinking under stress is only possible to a very limited extent; nevertheless, standardized checklists in such situations may help reduce further errors [3,7].

Numerous decision aids (e.g., Detect, Estimate, Set safety objectives, Identify, Do, Evaluate (DESIDE), Problem identification, Acquire information, Survey strategy, Select strategy (PASS), Situation, Options, Act, Repeat (SOAR), and Question, Promote Ideas, Decide, Review (QPIDR)) have been developed and implemented with considerable success. In aviation, the best-known model is undoubtedly the FOR-DEC model [6,8,9]. Based on this model, we developed a decision-making aid for medical emergency contexts. Our investigation with paramedic students showed that a brief training session promoted further understanding of structured decision-making and its proper implementation. The clearest increase in the training scenarios could be seen in the risk assessment, as well as in the question of their own competence or their permissions. This also correlates with the subjective assessment of the participants. Since contraindications are initially prioritized over side-effects in a risk assessment, we encourage the use of risk assessments rather than clarifying potential alternatives. In fact, in time-critical situations in aviation, risks are weighed [10]; in this respect, it is gratifying to see the strongest growth in this area.

In terms of prior knowledge, 50% of participants claimed knowledge of decision aids and familiarity with the FOR-DEC model. Knowledge of CRM or principles of CRM was indicated by 90% of participants, and the question of whether decision aids can be applied, initially affirmed by 40% of participants, rose to 71.4% (*p* = 0.011) following the training session. There were no significant changes in the participants’ subjective assessments before and after the training. Their confidence in decision-making rose from 4.5 to 4.8 points (*p* < 0.394) and their reasoning for the decisions from 5.3 to 5.6 points (*p* < 0.081). Aspects, such as indications, options, and risks, changed significantly, from 5.4 to 6.1 points (*p* = 0.045). To consider my team partner’s notes and include my team partner in decision-making was high and remained unchanged following the training (6.8 to 6.8 points, *p* = 0.555).

It is surprising that, despite the small number of participants included in this study, the safety in the use of decision aids only came to 71.4%. While this was a significant increase from before the training, obviously not all participants could be reached. On the other hand, the points “decision” before and after the training were equally 100% fulfilled. This was largely due to the scenarios in which the students were forced to make decisions, as described examples in Table 4. No decision, in this case, would have meant that the scenario was not continued.

The result of the simulation also raises the question as to why the individual aspects could not be fully fulfilled despite training. The confidence in decision-making was not significantly increased (*p* < 0.394). Although theoretical training may ultimately prove too limited, the training gave the impression that the routine adoption of a structured decision-making model must first be developed and then standardized. Therefore, the FAR-BEK model has been included in the local SOP and is now regularly used in training, and rescue operations debriefings as well.

The FAR-BEK model, like other decision-making processes, fits perfectly into a team-time out or 10-for-10 [11]. Standardization also appears to be indispensable since intuitive, unsystematic, and individualized experiences are not sufficient grounds on which to base clinical decision-making [12].

Paramedic decision-making is extremely context-sensitive and requires flexible cognitive skills and rapid adaptation to shifting priorities [12]. Further studies could also analyze the influence of cognitive characteristics on the use of decision aids. Because these types of decisions involve a complex and dynamic interaction between the environment, the patient, the available resources, and the experience and knowledge of the paramedic, their respective influence and reciprocal benefits within the context of decision support should be further analyzed.

This study is limited in its ability to show how decision-making processes are enacted in real patient care or rescue operations and in its ability to evaluate long-term effects; longitudinal investigations may prove useful in this regard. Furthermore, interpretation is limited by failure to consider how older, more experienced employees make decisions, focusing instead solely on student decision-making. However, internal quality assurance assessments suggest that young paramedics with little work experience, in particular, are more likely to make decisions without adequate reflection on associated risks and alternatives, whereas older paramedics are better able to reflect on these factors.

## 5. Conclusions

With a short training session, the awareness and the implementation of a structured decision-making process by paramedic students can be significantly increased. Nevertheless, no statement can be definitively made about its implementation in real patient care. The application of structured decision-making tools may need to be further consolidated for routine application.

## Figures and Tables

**Table 1:** Local competencies of trained and certified paramedics.

Category	Skills
Airway management	Bag-valve-mask ventilation, i-gel (supraglottic airway device), non-invasive ventilation, oxygen therapy
Pharmacotherapy (Mostly used if an emergency physician was requested—or is en route—to the emergency site)	Crystalloid infusion, acetylsalicylic acid, epinephrine, amiodarone, atropine, butylscopolamine, dimethindene maleate, flumazenil, furosemide, glucose, ipratropium bromide, heparin, ketamine, morphine, naloxone, midazolam, nitroglycerin, prednisolone, salbutamol, tranexamic acid, urapidil
Electrotherapy (Mostly used if an emergency physician was requested—or is en route—to the emergency site)	Defibrillation, cardioversion, transcutaneous pacing
Trauma	Pelvic sling, tourniquet, hemostatic gauze, needle decompression, spinal immobilization, bandages
Routes of administration	Intravenous, intramuscular, intraosseous, inhalation

**Table 2:** Topics which were taught during the training in addition to FAR-BEK (German abbreviation for facts, alternatives, risks, competence, decision, control).

Subjects of the Training
● Memory and cognitive performance: While under normal circumstances about 7 ± 2 units of information (“chunks”) are simultaneously and callable noticeable, under stress, the brain’s short-term memory capacity decreases substantially. ● Decision-making under stress: The non-reflective, intuitive process is activated, with no conscious decision-making process beforehand. This corresponds to the archaic fight or to the escape reaction. ● Fixation errors and consequences: Since intuitive decision-making under stress is often based on experience, there is a risk of fixation errors. The decision-maker sticks to an impression, loses the overall view and professional objectivity, so that alternatives or risks are misjudged (example: chest pain can only be an acute coronary syndrome). ● Influences on cognitive performance: Being short of sleep, fatigue, dynamic progress of the emergency, action pressure, etc. ● Influence of personal competence: Concerns the point “Ability/Authorization/Competence”. Reflection of one’s own professional capacity and competence. Cross-check with the team partner, 4 eyes principle, know your own weaknesses, own training state of medical skills, knowledge of medical facts and guidelines, etc. ● Examples of decisions from other disciplines: Aircraft ditching in the Hudson River, the sinking of the Costa Concordia, etc. ● Legal aspects of decisions: Those who act beyond their approvals must justify their measures. If an emergency exists, the ‘offender’ may take an action that is necessary, proportionate, and appropriate to avert the danger. In this respect, the paramedic must consider the urgency of the measures, as well as the distance to the nearest appropriate hospital, or the arrival time of the next emergency physician. ● Explanation of existing decision-making aids: For example DESIDE (Detect, Estimate, Set safety objectives, Identify, Do, Evaluate), FOR-DEC (Facts, Options, Risks & Benefits, Decision, Execution, Check), PASS (Problem identification, Acquire information, Survey strategy, Select strategy), SOAR (Situation, Options, Act, Repeat), etc.

**Table 3:** The FAR-BEK model (German abbreviation for facts, alternatives, risks, competence, decision, control) adapted from the FOR-DEC (Facts, Options, Risks and Benefits, Decision, Execution, Check) model.

FOR-DEC	FAR-BEK	Commentary
Facts	Fakten (facts)	Situation, facts, actual analysis (e.g., what is the correct indication for treatment?)
Options	Alternativen (alternatives, options)	Which options are available? Are less invasive measures possible? Have basic measures been completely carried out?
Risks	Risikoabwägung (risks)	Consideration of risks and benefits. Which risks and benefits are associated with the respective options for action?
Decision	Befugnis/Kompetenz (ability, authorization, competence)	Have I mastered the measure? Do I have approval for it? Do I need more resources, such as physicians, fire department, or a team for extracorporeal life support (ECLS)?
Execution	Entscheidung (decision)	Determine a method with the greatest chance of success and lowest associated risks. Perform the procedures (e.g., application according to the four-eye principle).
Control	Kontrolle (control)	Review of the implemented measure, monitoring, etc.

**Table 4:** Case studies from the training.

**Example 1: Focus Alternatives and Risks**
Unconscious patient (GCS 6) with suspected stroke or intracranial hemorrhage at compromised ventilation in a third-floor apartment. Due to the unconsciousness and need for emergency anesthesia with endotracheal intubation for transport, an emergency physician is requested to the site. However, this takes 20 min to the scene. Due to the circumstances of the scene, the only way out remains through the narrow stairwell. Decision making: Staying in the apartment with the advantage to carry out an adequate assisted ventilation under suction, but thereby to extend the rescue time. Or to carry out rapid transport through the stairwell with the advantage of initiating the transport and to approach the emergency physician in order to reach the target clinic more quickly. The risk of unmanageable vomiting and limited ventilation options is accepted.
**Example 2. Focus Competence and Approval**
Pregnant patient (36th week of pregnancy) with a painful radius fracture and necessary analgesia. Decision making: Physical measures (cooling, splinting) bring only limited relief so that pharmacological therapy seems inevitable. The paramedics have approval for ketamine for analgesia, which, however, is not recommended for analgesia in monotrauma in pregnant women. Or, as opiates might be indicated as potent analgesics, but for which the paramedics have no approval, to request the emergency physician to the scene.

## References

[1] Croskerry P. (2002). Achieving Quality in Clinical Decision Making: Cognitive Strategies and Detection of Bias. Acad. Emerg. Med..

[2] Meindl-Fridez C., Breckwoldt J., Battegay E. (2018). Medizinische (Notfall-)Entscheidungen und wie man Fehler vermeidet. Notfall Rettungsmed..

[3] Vetter B., Gasch B., Padosch S.A. (2015). Medizinisches Handeln in komplexen Notfallsituationen: Kompetent und erfolgreich entscheiden, kommunizieren und führen. Anaesthesist.

[4] Bubb H. (2005). Human reliability: A key to improved quality in manufacturing. Hum. Factors Man..

[5] Rall M., Lackner C.K. (2010). Crisis Resource Management (CRM). Notfall Rettungsmed..

[6] Soll H., Proske S., Hofinger G., Steinhardt G. (2016). Decision-Making Tools for Aeronautical Teams: FOR-DEC and Beyond. Aviat. Psychol. Appl. Hum. Factors.

[7] Arriaga A.F., Bader A.M., Wong J.M., Lipsitz S.R., Berry W.R., Ziewacz J.E., Hepner D.L., Boorman D.J., Pozner C.N., Smink D.S. (2013). Simulation-based trial of surgical-crisis checklists. N. Engl. J. Med..

[8] O’Hare D., Tsang P.S. (2003). Aeronautical Decision Making: Metaphors, Models, and Methods. Principles and Practice of Aviation Psychology.

[9] Hörmann H.J. FOR-DEC—A Prescriptive Model for Aeronautical Decision Making. Presented at the 21. WEAAP-Conference.

[10] Burger K.H. (2007). Basic Competence for Optimum Performance. Competence Criteria for Lufthansa Flight Crew Members.

[11] Rall M., Glavin R.J., Flin R. (2008). The ‘10-seconds-for-10-min principle’. Bull. R. Coll. Anaesthetists.

[12] Reay G., Rankin J.A., Smith-MacDonald L., Lazarenko G.C. (2018). Creative adapting in a fluid environment: An explanatory model of paramedic decision making in the pre-hospital setting. BMC Emerg. Med..

